# Current status and influencing factors of care burden of pancreatic cancer caregivers under COVID-19

**DOI:** 10.3389/fpsyg.2022.1066278

**Published:** 2023-01-04

**Authors:** Qingmei Sun, Jiarong Li, Xiaoping Fang, Jie Jin, Lei Cui

**Affiliations:** Pancreas Center, The First Affiliated Hospital with Nanjing Medical University, Nanjing, China

**Keywords:** pancreatic cancer, new crown epidemic, family caregivers, care burden, influencing factors

## Abstract

**Objective:**

To explore the level of care burden and its influencing factors of caregivers of pancreatic cancer patients during hospitalization under the background of COVID-19.

**Methods:**

From September 2021 to December 2021, in Jiangsu Province Hospital, the convenience sampling method was used to investigate the care burden level of family caregivers of pancreatic cancer patients, and univariate and multivariate analysis methods were used to analyze the influencing factors. The survey tools included the General Information Questionnaire, the Family Caregiver Care Burden Scale, the Hospital Anxiety and Depression Scale, the Benefit Discovery Rating Scale, and the General Self-Efficacy Scale.

**Results:**

A total of 100 subjects were included in this study, of which 45% were male and 55% were older than 50 years. In the Context of COVID-19, the care burden of caregivers of pancreatic cancer patients was at a mild level, and the main influencing factors were family economic status (*p* < 0.001), anxiety and depression level (*p* < 0.001) and self-efficacy (*p* < 0.001).

**Conclusion:**

Medical staff should pay attention to the caregivers of pancreatic cancer with a heavy family burden, and pay attention to their anxiety and depression, and take corresponding measures to improve the self-efficacy of the caregivers, so as to reduce the care burden.

## Introduction

Pancreatic cancer is one of the common malignant tumors of the digestive system. In 2018, the number of new cases of pancreatic cancer worldwide reached 458,918, and the number of deaths reached 432,242, with the number of deaths approximately equal to the number of cases (Bray et al., 2018). The incidence of pancreatic cancer in China is also increasing year by year, rising from the tenth to the seventh among all cancers (Sun, D. et al., 2020). Pancreatic cancer has a poor prognosis, with a 5-year survival rate of only 2–9%, ranking the lowest among all cancer types. Moreover, the quality of life of pancreatic cancer patients is significantly lower than that of other cancer types, which not only brings serious impact to patients, but also brings great impact to caregivers (Chu et al., 2017; Mcguigan et al., 2018).

In the process of caring for pancreatic cancer patients, caregivers generally have different degrees of care load, which will not only reduce the quality of life of caregivers themselves, but also affect the quality of care and thus reduce the quality of life of patients (Ansari et al., 2016). After the massive outbreak of COVID-19 at the end of 2019, major hospitals across the country implemented closed management and implemented “one person, one companion” to control the epidemic, and were not allowed to change caregivers at will. Studies have found that the panic caused by the epidemic, the heavy care task, the worry caused by the change of patients’ condition, and the psychological pressure caused by the hospital environment have doubled the burden of family caregivers (Bauer et al., 2018; Cohen et al., 2020). However, few studies have investigated the burden level of caregivers of pancreatic cancer patients and its influencing factors under the background of normalized COVID-19 prevention and control.

Therefore, this study aimed to investigate the current situation of the care burden of the main caregivers of pancreatic cancer patients during the COVID-19 outbreak in Nanjing in September 2021 and explore its influencing factors, so as to provide a theoretical basis for reducing the care burden of the main caregivers of pancreatic cancer patients under the closed management of the epidemic.

## Materials and methods

### Patient recruitment and exclusion criteria

From September 2021 to December 2021, 100 family caregivers of pancreatic cancer patients were selected by convenience sampling in Jiangsu Provincial People’s Hospital as the research objects. Inclusion criteria: ① Family caregivers of pancreatic cancer patients diagnosed by clinicopathological results; ② The family member who was designated by the pancreatic cancer patient to take care of them the most during the epidemic period; ③ Informed consent and willingness to participate in this study. Exclusion criteria: ① Family caregivers accompanied by serious physical or psychological diseases, such as cancer or obvious depression; ② The caregiver’s care time is less than 3 days.

### General information questionnaire

The general information questionnaire was made by the researchers, including the patient’s name, age, gender, duration of illness, type of disease, caregiver’s age, gender, duration of care, educational background, family monthly income, number of children, relationship with the patient, work status, family economic pressure, etc.

### Caregiving burden scale

The Caregiver Burden Scale of Cancer Patients (CBS-CP) contains 31 items, the last two of which evaluate the overall burden. In this study, the first 29 items were used for investigation, which were divided into 5 factor dimensions: physical burden (factor 1), economic burden (factor 2), psychological burden (factor 3), social burden (factor 4) and disease concept burden (factor 5). The SCALE is scored 0 points for “NEVER,” 1 point for “occasionally,” 2 points for “sometimes,” 3 points for “often” and 4 points for “always.” The total score of burden ≤29 is no burden; 29 < total score ≤ 58 is considered as mild burden; 59 < total score ≤ 87 is considered as moderate burden; A total score of >87 is considered as severe burden. Cronbach’s α coefficient of CBS-CP total scale and 5 factors fluctuated between 0.70 and 0.96, indicating good reliability.

### Caregiver benefit discovery scale

The Caregiver Benefit Discovery Scale can be used to assess whether cancer patients and caregivers experience or benefit from positive aspects in coping with cancer challenges, including strengthened relationships with family members, personal growth as patients or caregivers, and healthier lifestyles inspired by cancer diagnosis and treatment (Li et al., 2017). The scale consists of 17 items on a 5-point scale, with 1 being “not at all” and 5 being “very much.” The higher the score, the more benefit the patient perceived on that item. Cronbach’s α coefficient of the overall benefit discovery scale and the three dimensions was ≥0.891, indicating good reliability.

### Hospital anxiety and depression scale

Hospital Anxiety and Depression Scale is mainly used for rapid assessment of patients’ anxiety and depression, and is one of the tools for screening anxiety and depression in clinical practice. The scale has 14 items, including 7 items for anxiety and 7 items for depression. Each item was scored at 4 levels from 0 to 3, with each subscale scoring from 0 to 21. The total score of 8–10 was classified as mild anxiety/depression, 11–14 as moderate anxiety/depression, and ≥ 15 as severe anxiety/depression (Bjelland et al., 2002).

### General self-efficacy scale

The General Self-efficacy Scale (GSES), compiled by German psychologist Ralf Schwarzwe (Schröder et al., 1997), consists of 10 items and is scored by the 4-point Likert Scale. “Completely incorrect” counts for 1 point, while “somewhat correct” counts for 2 points. “Mostly correct” counts for 3 points and “exactly correct” counts for 4 points. The higher the total score, the higher the self-efficacy of the subject. The Cronbach’s α coefficient of the scale was 0.87.

### Data collection methods and quality control

Data were collected by two professionally trained nurses. The General Information Questionnaire, Care Burden Scale, Caregiver Benefit Discovery Scale, and General Self-efficacy Scale were distributed to caregivers who met the inclusion criteria and agreed to participate in the study. The investigators handed out questionnaires on site, and used unified instructions to explain the significance of the survey, the time required and the notes for filling in the survey to the caregivers. For caregivers who were illiterate or had visual impairment, the investigator stated the questions and options of the questionnaire, and the researcher checked them after answering the questions. General information and disease-related information can be obtained from medical records. If there is no record in medical records, consult the medical staff in charge of the bed or the caregivers themselves. Collect and check the questionnaire on the spot, and check with caregivers in time to ensure the completeness and accuracy of the questionnaire.

### Statistical analysis

SPSS 22.0 software was used for data entry and statistical analysis. Measurement data were described by mean and standard deviation, and enumeration data were described by frequency and percentage. Independent sample t-test, ANOVA test, Pearson correlation analysis and multiple linear regression analysis were used to analyze the influencing factors of caregiver burden. In two-sided test, *p* < 0.05 was considered statistically significant.

## Results

**Figure 1:**
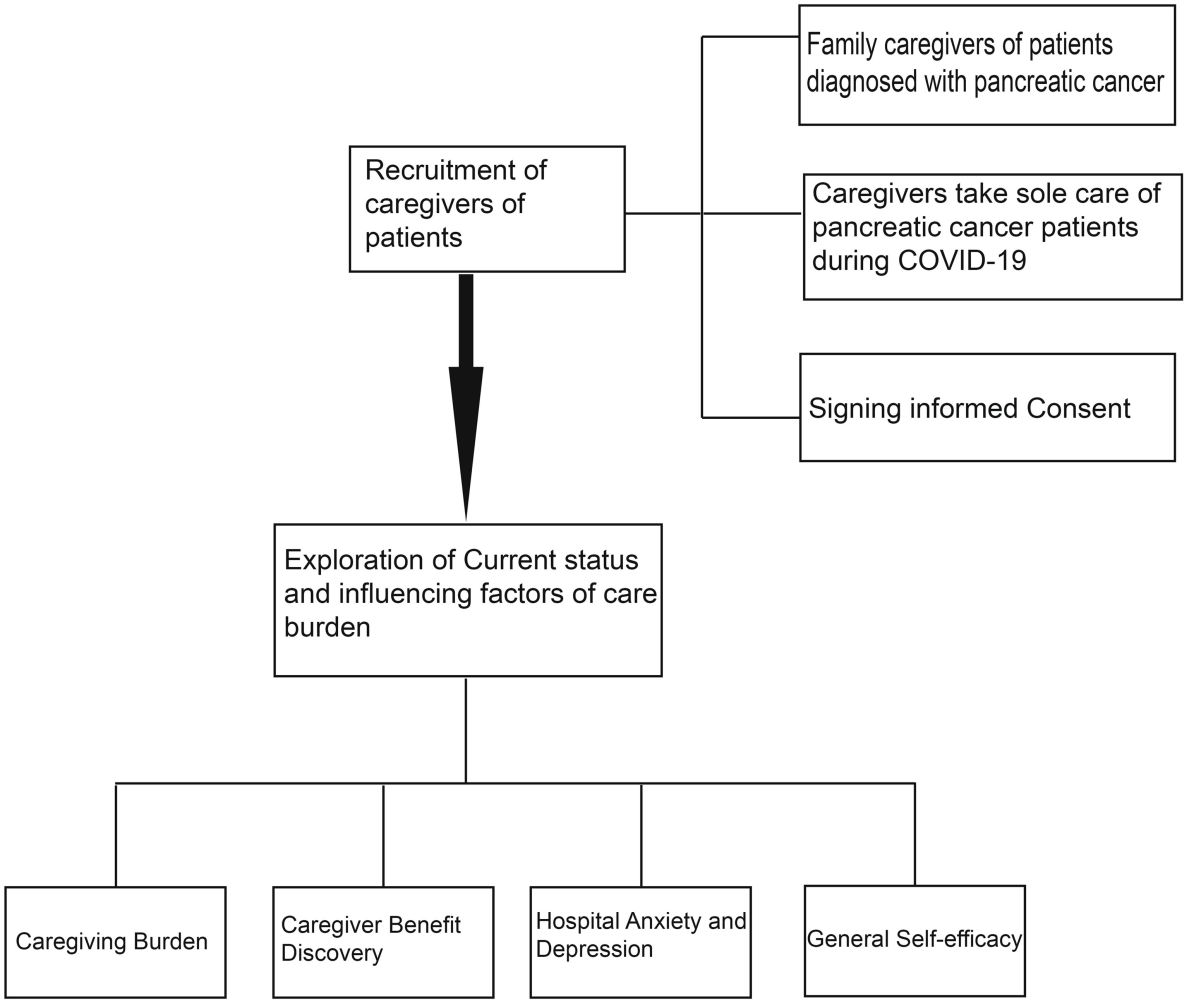
The flow chart.

Figure 1 shows our workflow.

### General information about caregivers

A total of 100 questionnaires were distributed and 100 were recovered, with a recovery rate of 100%. Among the 100 pancreatic cancer caregivers, 55% were older than 50 years and 45% were male. The rest of the general information is shown in Table 1.

**Table 1:** General Information Questionnaire of caregivers of pancreatic cancer (*n* = 100).

Indicators		The number of cases	Composition ratio (%)
Age(years)	<30	3	3.0
	31–40	19	19.0
	41–50	23	23.0
	51–60	27	27.0
	>60	28	28.0
Gender	Male	45	45.0
	Female	55	55.0
Level of education	Primary school and below	17	17.0
	High school	42	42.0
	Technical secondary school and junior college	20	20.0
	Bachelor degree or above	21	21.0
Monthly household income (yuan)	<3,000	14	14.0
	3,000–5,000	28	28.0
	>5,000	58	58.0
Relationship with patients	spouse	53	53.0
	children	43	43.0
	parents	2	2.0
	Brothers and sisters	1	1.0
	others	1	1.0
Medical insurance reimbursement rate	≤30%	6	6.0
	31–60%	25	25.0
	61–80%	54	54.0
	81–100%	15	15.0
Knowledge of the condition	Have no idea	2	2.0
	Know a little	31	31.0
	Probably know	46	46.0
	Know very well	21	21.0
Total care time	<1 month	63	63.0
	1-3 months	25	25.0
	3-6 months	2	2.0
	>6 months	10	10.0
Family financial pressure	The heavier pressure	36	36.0
	Moderate pressure	49	49.0
	Lighter pressure	15	15.0
Working state	on-the-job	44	44.0
	Not on-the-job	56	56.0

### Comparison of care burden scores of caregivers with different characteristics

The caring burden score of the main caregivers of pancreatic cancer patients was 44.04 ± 20.9, including 30 without burden, 43 with mild burden, 26 with moderate burden, and 1 with severe burden. There were statistically significant differences in the care burden of main caregivers with different ages, education levels, family monthly income, degree of understanding of illness, and family economic pressure (all *p* < 0.05; Table 2).

**Table 2:** Comparison of care burden scores of caregivers of pancreatic cancer patients with different characteristics (scores).

Indicators	Group	The assignment	Scores (^−^ x ±s)	t/*F* value	*p value*
Age (years)	<30	1	45.67 ± 20.65	3.338	0.013
	31–40	2	37.47 ± 18.74		
	41–50	3	44.43 ± 19.35		
	51–60	4	55.03 ± 22.81		
	>60	5	37.39 ± 18.25		
Gender	Male	1	40 ± 21	−1.748	0.084
	Female	2	47 ± 21		
Level of education	Primary school and below	1	60.76 ± 18.36	8.079	<0.001
	High school	2	46.23 ± 21.48		
	Technical secondary school and junior college	3	37.65 ± 17.01		
	Bachelor degree or above	4	32.19 ± 15.38		
Monthly household income (yuan)	<3,000	1	49.50 ± 25.10	4.068	0.020
	3,000–5,000	2	51.24 ± 20.71		
	>5,000	3	39.03 ± 18.76		
Relationship with patients	spouse	1	43.30 ± 20.18	0.706	0.590
	children	2	45.86 ± 22.27		
	parents	3	27.50 ± 0.70		
	Brothers and sisters	4	25.0		
	others	5	57.0		
Medical insurance reimbursement rate	≤30%	2	55.00 ± 27.72	1.267	0.290
	31–60%	3	46.48 ± 21.35		
	61–80%	4	43.68 ± 20.36		
	81–100%	5	36.87 ± 18.65		
Knowledge of the condition	Have no idea	1	42.50 ± 12.02	5.269	0.002
	Know a little	2	55.03 ± 19.19		
	Probably know	3	41.13 ± 20.22		
	Know very well	4	34.33 ± 19.45		
Total care time	<1 month	1	44.10 ± 21.46	0.161	0.922
	1-3 months	2	43.76 ± 18.27		
	3-6 months	3	35.00 ± 22.63		
	>6 months	4	46.30 ± 25.76		
Family financial pressure	The heavier pressure	1	56.12 ± 18.12	16.613	<0.001
	Moderate pressure	2	39.92 ± 19.67		
	Lighter pressure	3	24.17 ± 9.90		
Working state	on-the-job	1	44.36 ± 20.58	0.137	0.892
	Not on-the-job	2	43.78 ± 21.35		

### Benefit discovery, anxiety and general self-efficacy of caregivers of pancreatic cancer patients

The total score of anxiety and depression of caregivers of pancreatic cancer patients was 15.94 ± 9.64, the benefit finding was 80.8 ± 17.05, and the general self-efficacy was 25.14 ± 8.20. Pearson correlation analysis showed that benefit finding was inversely proportional to care burden (*r* = −0.558, *p* < 0.05), anxiety and depression were inversely proportional to care burden (*r* = 0.676, *p* < 0.05), and self-efficacy was inversely proportional to care burden (*r* = −0.280, *p* < 0.05), as shown in Table 3.

**Table 3:** Correlation analysis of care burden of caregivers of pancreatic cancer patients and its influencing factors (R value).

Indicators	Care burden
Benefits found	−0.558^*^
Anxiety depression	0.676^*^
Self-efficacy	−0.280^*^

* *P* < 0.05.

### Multiple linear regression analysis of influencing factors of care burden of caregivers of patients with pancreatic cancer

In patients with pancreatic cancer burden of caregivers to take care of the total score as the dependent variable, the single factor analysis was statistically significant in the project as independent variables, multivariate linear regression analysis, finally found that anxiety depression (*p* < 0.001), and family economic pressure (*p* = 0.001) and self-efficacy (*p* = 0.014) are the influence factors of pancreatic cancer caregiver burden of care, The specific results are shown in Table 4.

**Table 4:** Multiple linear regression analysis of influencing factors of care burden of caregivers of pancreatic cancer patients.

The independent variables	B value	SE value	Β-value	T-value	*p-value*
Constant	36.138	16.250	–	2.224	0.029
Age	−0.82	1.392	−0.005	−0.059	0.953
Knowledge of the condition	−1.097	2.255	−0.040	−0.486	0.628
Monthly household income	1.24	2.163	0.043	0.573	0.568
Level of education	−1.277	1.831	−0.062	−0.697	0.487
Sense of benefit from illness	−1.04	0.089	−0.085	−1.171	0.245
Anxiety depression	0.896	0.177	0.414	5.052	<0.001
Family financial pressure	8.766	2.257	0.280	3.400	0.001
Self-efficacy	−0.544	0.218	−0.210	−2.502	0.014

## Discussion

The results of this study showed that the total score of care burden of caregivers of pancreatic cancer patients was (44.04 ± 20.9), which was generally at a mild level, among which 30% reported no burden, 43% reported mild burden, 26% reported moderate burden, and 1% reported severe burden. This finding is consistent with that of caregivers of breast and lung cancer patients in China, which cannot be compared with the burden of care in a non-pandemic setting due to the lack of research on caregivers of pancreatic cancer patients (Borges et al., 2017; Sun et al., 2019). Previous studies conducted qualitative interviews on caregivers of patients with inoperable pancreatic cancer, and the results showed that caregivers’ burden experience mainly included psychological burden, physical burden, lack of knowledge and emotional support, challenges in personal life, burden of disease notification and end-of-life decision-making (Xia et al., 2022). Pancreatic cancer has no characteristic symptoms in the early stage, and most patients are already in the late stage when they are diagnosed. The sudden disease will lead to the responsibility of caring for the family members in the case of insufficient preparation. At the same time, during the epidemic prevention and control period, the hospital’s closed management measures prohibit the random change of caregivers, resulting in all the care pressure concentrated in one person. This caregiver not only faces the stress of caring for the disease, but also faces the risk of the epidemic, which can easily lead to negative emotions and even physical health problems. It is necessary to pay attention to the physical and mental health of caregivers of pancreatic cancer (Sun et al., 2019).

Most of the pancreatic cancer patients in this study rated their family financial stress as moderate (49%) or heavy (36%), and the greater the family financial stress, the heavier the care burden of pancreatic cancer caregivers. Due to the continuous development and improvement of tumor treatment technology, while prolonging the survival time of patients, it also brings some economic burden to families, including high medical expenses, care costs and income reduction caused by the reduction of labor force. Some studies have shown that the family income of cancer patients after the disease is 32% lower than that before the disease, and the annual medical expenditure of cancer patients is 1.18 times of the average annual expenditure of the family (Xia et al., 2022). Pancreatic cancer mainly occurs in middle-aged and elderly people. The middle-aged people are the main labor force of the family, and their normal work is affected by the disease, which affects their family income (Khalaf et al., 2021). The elderly, due to physical reasons, have underlying diseases or are prone to complications, and so on, resulting in more follow-up treatment and care expenses (Huang et al., 2021). In addition, during the closed management of the epidemic, it greatly affected the normal work of caregivers and affected their economic income (Klein, 2021). Heavy economic burden will not only affect the treatment choice of patients, but also lead to patients’ quality of life decline, psychological distress and behavior change and many other adverse outcomes. Because the psychological burden perception of caregivers is closely related to the patient’s own condition, it will eventually lead to the increased perceived burden of caregivers. Therefore, in future studies, we should focus on patients and caregivers with heavy family burden, and actively carry out education, counseling and intervention programs for high-risk groups, so that caregivers can make full use of family and social resources and reduce the care burden caused by economic pressure.

In this study, the anxiety and depression score of caregivers was (15.94 ± 9.64), which was relatively severe. The anxiety and depression was directly proportional to the care burden. This is in line with the findings of a study on the quality of life of caregivers of pancreatic cancer patients, which showed that 14 to 32 percent of caregivers met the clinical cutoff for depression. Compared with patients with pancreatic cancer, caregivers had a significantly higher proportion of negative emotions such as anxiety and depression, which may be due to more stress on caregivers, such as bathing, feeding, wound/drainage care and monitoring complications. At the same time, the severe epidemic situation and the stress and pressure caused by the closed management of hospitals are more likely to lead to mental health problems of caregivers. Adverse emotional responses of caregivers can lead to a series of negative effects, such as the decline of their own health, poor quality of care, and emotional problems of patients (Bevans and Sternberg, 2012; Litzelman et al., 2016). In the actual process of clinical nursing, medical staff often ignore the psychological status of caregivers. In future research, it is necessary to evaluate the psychological status of patients and caregivers at the same time, and treat both sides as a whole. A cohort study showed that caregiver burden was primarily related to the baseline psychosocial health status of caregivers and patients (Jansen et al., 2021). Therefore, in future research, it is necessary to identify caregivers with poor psychological status early, so as to focus and target interventions.

Self-efficacy is an individual’s subjective judgment of his ability to perform a certain behavior, that is, his confidence in his ability to perform a certain behavior and achieve the expected results. For caregivers, it is a prerequisite to implement better care behaviors. Higher self-efficacy can help caregivers cope with care tasks with more confidence and less negative emotions, so as to cope with illness stress. This study found that the lower the self-efficacy of pancreatic cancer caregivers, the higher the care burden, which is consistent with previous studies. At present, a number of studies have been conducted to intervene the self-efficacy of cancer caregivers, and all of them have achieved good results. Hendrix conducted a randomized controlled trial of self-efficacy and care stress in caregivers of cancer patients, and the results showed that the intervention group had greater improvements in self-efficacy in cancer symptom management, stress management, and care preparation than the control group (Hendrix et al., 2016). A study on the influencing factors of the self-efficacy of caregivers of cervical cancer patients showed that caregivers with the number of shared help caregivers had higher self-efficacy than caregivers without the number of shared help caregivers. Due to the inability to change caregivers during the pandemic, one person must assume the care task, which may affect the self-efficacy of caregivers and ultimately increase their care burden. Therefore, in clinical nursing, it is necessary to pay attention to caregivers who have long periods of solitary care, screen and assess their perception of care burden as early as possible, and take corresponding intervention measures (Zou et al., 2021a,b).

So far, some studies have revealed the psychological impact of COVID-19 on care givers. Sun et al. found that in the early stage, negative emotions dominated, and self-treatment styles and psychological growth played an important role in maintaining the mental health of nurses (Sun, N. et al., 2020b). Panda et al. (2021) found that similarly, 52.3 and 27.4% of caregivers had anxiety and depression, respectively, during their isolation from their children (). Our study found that the caregiver burden of pancreatic cancer patients was at a mild level, and the main influencing factors included family economic pressure, caregiver anxiety and depression, and self-efficacy. These results can provide reference for psychological counseling of pancreatic cancer caregivers.

## Conclusion

In conclusion, the care burden of caregivers of pancreatic cancer patients is at a mild level, and the main influencing factors include family economic pressure, anxiety and depression of caregivers and self-efficacy. In order to reduce the care burden of caregivers, but also to further improve the quality of care, medical staff should timely psychological counseling for caregivers with negative emotions, early attention should be paid to caregivers with poor family economic status and low self-efficacy, and carry out disease education for them. In future studies, we should expand the sample size and collect multi-center data to more comprehensively explore the influencing factors, so as to construct targeted intervention programs.

## Data availability statement

The original contributions presented in the study are included in the article/supplementary material, further inquiries can be directed to the corresponding author.

## Ethics statement

The studies involving human participants were reviewed and approved by The First Affiliated Hospital of Nanjing Medical University. The patients/participants provided their written informed consent to participate in this study.

## Author contributions

QS and XF designed the study and were responsible for the submission of the final version of the paper. QS, JJ, and JL were involved in database search and statistical analyses. QS and JC were involved in the writing of manuscript and its critical revision. All authors contributed to the article and approved the submitted version. All authors agree to be accountable for all aspects of the work.

## Conflict of interest

The authors declare that the research was conducted in the absence of any commercial or financial relationships that could be construed as a potential conflict of interest.

## Publisher’s note

All claims expressed in this article are solely those of the authors and do not necessarily represent those of their affiliated organizations, or those of the publisher, the editors and the reviewers. Any product that may be evaluated in this article, or claim that may be made by its manufacturer, is not guaranteed or endorsed by the publisher.
